# Ruptured Uterine Leiomyosarcoma With Heterologous Components Including Osteosarcoma and Chondrosarcoma

**DOI:** 10.14740/jmc5266

**Published:** 2026-03-04

**Authors:** Takuma Hayashi, Yasuaki Amano, Ikuo Konishi

**Affiliations:** a Cancer Medicine, National Hospital Organization Kyoto Medical Center, Kyoto-city, Kyoto 612-8555, Japan; b Department of Obstetrics and Gynecology, Kyoto University Hospital, Kyoto-city, Kyoto 606-8507, Japan

**Keywords:** Leiomyosarcoma, Osteosarcoma, Chondrosarcoma, Ruptured uterine leiomyosarcoma

## Abstract

Tumor rupture is a rare complication of uterine leiomyosarcoma. We report a case of ruptured uterine leiomyosarcoma diagnosed after the onset of abdominal pain following endoscopic examination of the lower gastrointestinal tract. The patient was a 56-year-old woman who was diagnosed with anemia at 48 years of age when she was first referred to our medical team. Contrast-enhanced magnetic resonance imaging (MRI) revealed a 48 × 50 mm mass in the anterior wall of the uterine body, which was diagnosed as a uterine fibroid. After 8 years of regular follow-up once or twice a year, the patient developed abdominal pain after undergoing lower gastrointestinal endoscopy, which was prompted by a positive fecal occult blood test result. Contrast-enhanced computed tomography (CT) showed that the uterine mass had enlarged to 90 × 69 mm. T2-weighted contrast-enhanced MRI demonstrated moderate signal intensity and restricted diffusion, whereas contrast-enhanced T1-weighted MRI revealed high signal intensity, suggestive of hemorrhage. The outlines of the tumor and uterus were interrupted cephalad to the lesion, raising the suspicion of rupture of a malignant uterine mesenchymal tumor. Therefore, total hysterectomy, bilateral salpingo-oophorectomy, and partial omentectomy were performed. Intraoperatively, tumor rupture and adhesion of the ruptured tissue to the ileum were observed, necessitating partial ileectomy. Pathological examination of the resected specimen revealed irregularly proliferating spindle cells with marked nuclear atypia, 12 mitotic figures per 10 high-power fields, coagulative necrosis, and multinucleated giant cell infiltration. Another notable finding was the presence of ectopic osteosarcoma and chondrosarcoma.

## Introduction

In clinical practice, uterine leiomyosarcoma is relatively rare, but is known to have a poor prognosis. Few reports have described uterine leiomyosarcomas with ectopic components. As a result, the malignancy and clinical impact of uterine leiomyosarcoma with ectopic components remain unclear. Ovarian tumor rupture is relatively common in clinical practice, whereas uterine tumor rupture is rare. In this case, the patient developed abdominal pain after endoscopic examination of the lower gastrointestinal tract and was diagnosed with ruptured uterine leiomyosarcoma containing ectopic components. Here, we report this case, along with a review of the literature.

## Case Report

The patient was a 56-year-old woman (height: 156 cm, weight: 54 kg) with a medical history of total hip replacement for congenital hip dislocation at age 53 and a family history of a father with subarachnoid hemorrhage and a mother with gastric cancer. The patient’s menstrual history included menarche at the age of 13 and menopause at 55. She had no previous pregnancy or delivery. Her chief complaint relating to the pregnancy was abdominal pain. The patient did not take any medication.

### History of present illness

**Figure 1:**
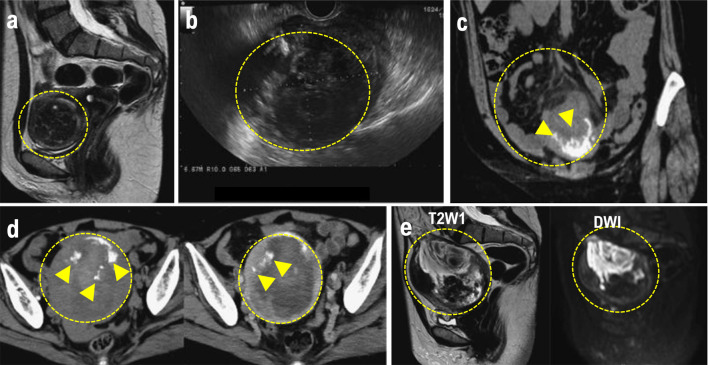
(a) MRI performed at the initial consultation. The mass exhibited a clearly demarcated low signal intensity on T2W1 and was diagnosed as a uterine fibroid. (b) Transvaginal ultrasound after lower gastrointestinal endoscopy revealed a 90 × 69 mm mass in the uterine body, with a heterogeneous interior and irregular margins. (c) Plain CT. Increased density was observed in the mesenteric fat tissue surrounding the mass. A mass with marginal calcification was found on the anterior wall of the uterine body, protruding cephalad. (d) Comparison of the initial CT (left) with a CT performed 8 days later for preoperative evaluation (right). The uterine mass had shrunk from 10 × 11 cm (left panel) to 10 × 9.5 cm (right panel), and the absorption value of the internal high-density area had decreased, suggesting absorption of the hematoma. (e) MRI for further investigation of suspected uterine sarcoma. The mass showed moderate signal intensity on T2W1 and high signal intensity on DWI, and heterogeneous signals were present within it, suggesting hemorrhage and necrosis. The mass is indicated by the yellow dotted circle. Heterologous components including osteosarcoma and chondrosarcoma are indicated by the white arrowheads. CT: computed tomography; MRI: magnetic resonance imaging.

At 48 years of age, the patient was diagnosed with anemia (Hb 8.8 g/dL). As a result, she was referred to our gynecological clinic for further evaluation to determine the presence of a gynecological disease. Transvaginal ultrasonography revealed a large mass (48 × 50 mm) compressing the lining of the anterior uterine wall. Based on the contrast-enhanced magnetic resonance imaging (MRI) findings, the mass was diagnosed as a uterine leiomyoma (Fig. 1a). However, because the patient preferred monitoring until menopause, we prescribed iron supplements to treat the anemia and continued tumor follow-up. The tumor did not exhibit any signs of enlargement. At 56 years of age, however, she developed lower abdominal pain after undergoing an endoscopic examination of the lower gastrointestinal tract. Blood tests revealed an elevated C-reactive protein (CRP) level (8.49 mg/dL). We suspected peritonitis due to delayed perforation and performed contrast-enhanced computed tomography (CT). Imaging revealed increased adipose tissue density in the mesenteric lining around the uterus and ascites.

The findings resulting from a physical examination were as follows: blood pressure, 135/72 mm Hg; pulse, 64 beats/min; mild abdominal tenderness. Pelvic examination revealed a small amount of white vaginal discharge, erosions in the uterine vaginal region, a first-sized and mildly tender uterine body, and a non-palpable adnexa. Transvaginal ultrasonography revealed a 90 × 69 mm heterogeneous mass with irregular margins on the uterine body (Fig. 1b). Blood tests revealed no abnormalities except for an elevated platelet count of 500,000/µL. The liver and renal function test results were normal. The serum lactate dehydrogenase (LDH) and total protein (TP) levels were elevated to 401 U/L and 8.2 g/dL, respectively. The fibrinogen level was 604 mg/dL, indicating coagulation abnormalities. The CRP level was 0.37 mg/mL, slightly above the normal range. The tumor marker levels were within normal limits: CEA 1.8 ng/mL, CA19-9 9 U/mL, and CA125 18 U/mL. Endoscopy of the lower gastrointestinal tract showed no abnormal findings. Truncal CT (Fig. 1c) revealed a 10 × 11 cm mass with marginal calcification on the anterior uterine wall and cranial protrusions of the uterus. A pale, low-density area suggestive of a hemorrhage was observed within the mass, and a small amount of pelvic fluid was present, indicative of the presence of blood. The increased adipose tissue density surrounding the mass was suggestive of a possible rupture.

A contrast-enhanced CT performed for preoperative evaluation 8 days after the initial scan showed that the uterine mass had decreased to 9.5 × 9.5 cm and the bloody ascites had resolved (Fig. 1d). The fat density surrounding the cranial protrusion of the uterus decreased, and the wall in that region thinned. The low-density background area within the mass became less prominent, indicative of a reduction in bloody fluid. No lymph node enlargement or distant metastasis was observed. Pelvic contrast-enhanced MRI (Fig. 1e) showed a 10-cm mass located within the muscle layer of the anterior uterine wall. T2W1 demonstrated moderate signal intensity within the mass and DWI demonstrated high signal intensity. Heterogeneous internal signals suggestive of hemorrhage and necrosis were observed. T2W1 also showed signal imaging, which showed a low signal area on the ventral side of the mass. CT revealed areas of calcification, and contrast-enhanced MRI confirmed that both ovaries were normal. Based on the rapid tumor growth accompanied by bleeding, necrosis, rupture, and elevated LDH levels, rupture of the uterine leiomyosarcoma was suspected. The preoperative stage corresponded to stage IB disease.

### Surgical findings

A rupture of the tumor mass on the anterior uterine wall was observed, with the hematoma and tumor contents exposed in the abdominal cavity. Adhesion between the tumor and ileum was observed near the rupture site, raising the suspicion of tumor infiltration. Macroscopically, both the adnexa were normal. No lesions suggestive of disseminated metastasis were identified in the abdominal cavity; therefore, a simple total hysterectomy, bilateral salpingo-oophorectomy, partial omentectomy, or partial ileectomy were performed.

### Histopathological findings

**Figure 2:**
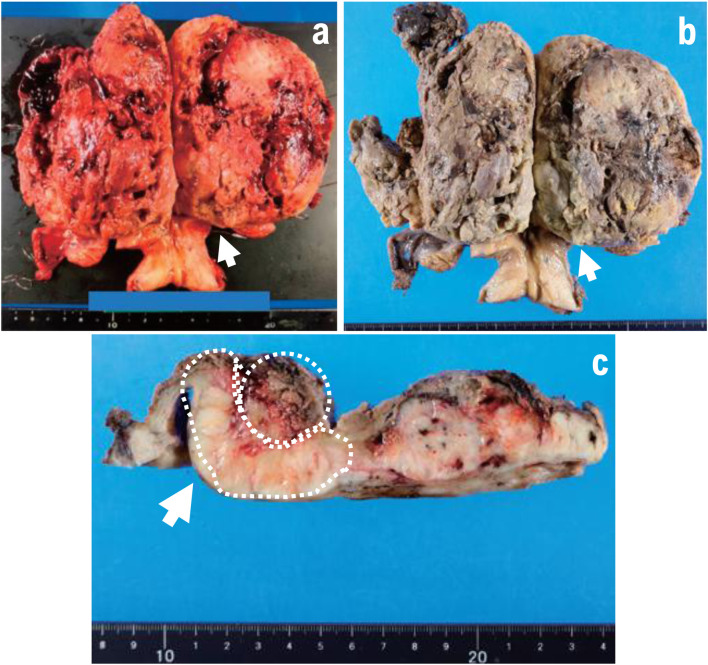
(a–c) Excised specimen. Uterine adnexa, 480 g. A 12-cm mass lesion accompanied by bleeding was observed in the uterine body (arrow). A cross-section of the tumor after fixation revealed bleeding within a slightly shiny yellowish-white solid tumor. The mass is indicated by the yellow dotted circle. Heterologous components including osteosarcoma and chondrosarcoma are indicated by the white arrowheads.

**Figure 3:**
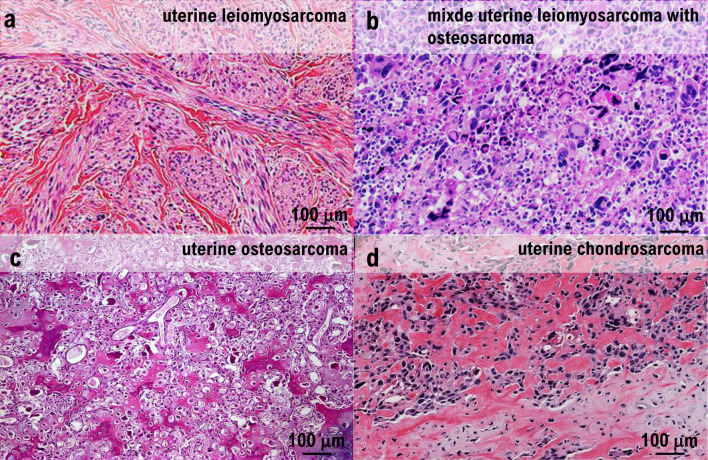
Histopathological examination. (a) Irregular fascicular proliferation of spindle cells with prominent nuclear atypia was observed. Coagulative necrosis was also observed. (b) Approximately 12 mitotic figures were observed per 10 high-power fields. (c) Polygonal giant cells were observed throughout. (d) Ectopic elements of osteosarcoma and chondrosarcoma were present.

A 12-cm hemorrhagic lesion was identified in the uterine body (Fig. 2a–c). Histologically, an irregular fascicular proliferation of spindle cells with marked nuclear atypia was observed. Approximately 12 mitotic figures per 10 HPF and areas of coagulation necrosis were observed, supporting the diagnosis of uterine leiomyosarcoma (Fig. 3a, b). Multinucleated giant cells were scattered throughout the tumor tissue (Fig. 3c), and ectopic osteosarcomatous and chondrosarcomatous elements were identified (Fig. 3d). Ectopic bone and cartilage elements were extensively observed in 29 of 33 pathological sections of the uterine leiomyosarcoma. A 12-cm mass lesion accompanied by hemorrhage was observed in the uterine body (Fig. 2a–c).

No neoplastic changes are observed in the endometrium. Benign leiomyomas were not observed. The Ki67 labeling index was 50%. No evidence of tumor invasion was found in the ileum, which was attached to the tumor and resected (Supplementary Material 1, jmc.elmerpub.com). Surgical pathological examination of the excised tissue revealed that the uterine leiomyosarcoma invaded the veins (Supplementary Material 2, jmc.elmerpub.com). As a result, metastatic lesions may be found in the lungs or liver within 2 years of surgical treatment. No malignant findings were observed in the cervix, bilateral adnexa, or greater omentum. Based on these histological and pathological findings, the patient was diagnosed with a ruptured uterine leiomyosarcoma with ectopic components (pT1bN0M0).

### Postoperative course

In this case, rupture of the uterine leiomyosarcoma was observed, and the medical staff determined that the risk of recurrence and metastasis was high. Therefore, we administered six cycles of doxorubicin as postoperative adjuvant chemotherapy. Contrast-enhanced CT performed after the completion of adjuvant therapy revealed no evidence of recurrence. However, a contrast-enhanced CT scan performed 1 year after surgery revealed a 9–12 mm mass in the right upper lobe of the lung (Supplementary Material 3, jmc.elmerpub.com). Partial thoracoscopic resection was performed, and pathological examination confirmed that the mass was a metastatic uterine leiomyosarcoma with osteosarcoma and chondrosarcoma.

## Discussion

Leiomyosarcomas with ectopic components are extremely rare, even when an extrauterine origin is considered, and uterine leiomyosarcomas with ectopic components arising from the uterus are particularly uncommon [1–3]. Examples of sarcomatous lesions with ectopic components include osteosarcoma, angiosarcoma, rhabdomyosarcoma, and liposarcoma [4]. The uterine leiomyosarcoma in this case contained osteosarcoma and chondrosarcoma components. Among previously reported cases of sarcomas with ectopic components, there have been few cases of uterine leiomyosarcoma with osteosarcomatous and chondrosarcomatous elements [5–8]. To the best of our knowledge, only seven such cases have been reported to date.

As uterine leiomyosarcomas containing ectopic components have been reported to originate from other organs, medical staff should perform full-body assessments at the time of diagnosis to determine whether lesions are present in other organs [3]. In this case, imaging studies revealed no extrauterine lesions, and the tumor was considered to be primary to the uterus. Based on imaging and pathological findings, the patient was diagnosed with uterine leiomyosarcoma containing rare osteosarcomatous and chondrosarcomatous components.

This case was accompanied by the rupture of a uterine leiomyosarcoma. The rupture of uterine tumors is rare, and only four cases of uterine leiomyosarcoma rupture have been reported to date [9–12]. In the literature, uterine fibroid rupture has been attributed to causes including traffic injuries and post-defecation events [13].

However, uterine leiomyosarcoma rupture may also occur because of tumor growth [9] or abdominal pressure, such as during defecation [10]. In the present case, the tumor was discovered when the patient developed abdominal pain immediately after endoscopic evaluation of the lower gastrointestinal tract; therefore, rupture due to external stimuli was suspected. However, in a previous routine examination, no significant increase in tumor size was noted, suggesting rapid interval growth. Exogenous uterine rupture generally does not occur without substantial external forces, such as trauma.

Although there have been reports of iatrogenic rupture during intrauterine balloon insertion for postpartum hemorrhage [14], there are currently no reports describing its use in non-pregnant women. It is highly likely that the primary cause of uterine leiomyosarcoma rupture is rapid tumor growth. The high Ki67 labeling index of the tumor is also indicative of rapid proliferation.

Uterine leiomyosarcoma has a poor prognosis, with a 5-year survival rate of 40–70% for stage I–II tumors [1]. In this case, because the tumor had ruptured, the medical staff believed that the risk of recurrence was higher than that for typical stage I tumors and therefore administered postoperative chemotherapy. However, despite the completion of chemotherapy, the patient developed recurrence and metastasis. Although the impact of rupture on the prognosis of uterine leiomyosarcoma remains unclear, rupture has been reported to be an independent poor prognostic factor in gastrointestinal stromal tumors, which are malignant stromal tumors originating from the gastrointestinal tract [15]. Thus, ruptured uterine leiomyosarcomas may also have a poor prognosis. In addition, reports have indicated that leiomyosarcomas containing ectopic components originating from other sites have a poor prognosis [16], and that osteosarcomas and chondrosarcomas arising in the uterus also show poor outcomes [17]. However, whether uterine leiomyosarcomas with ectopic components have a worse prognosis than ordinary uterine leiomyosarcomas remains uncertain. In this case, no recurrence was observed on contrast-enhanced CT at the completion of adjuvant chemotherapy; however, lung metastases were detected 1 year after surgery, suggesting high tumor malignancy and limited benefit from postoperative adjuvant chemotherapy.

Unlike uterine leiomyomas, uterine leiomyosarcomas can develop after menopause, even in women aged up to 80 years. The 5-year survival rate of patients with uterine leiomyosarcomas is less than 20% [18]. Therefore, an early diagnosis and surgical treatment are essential for long-term survival.

### Conclusion

Uterine leiomyosarcoma with ectopic components is rare. Therefore, the clinical picture of this type of tumor remains largely unknown. However, the clinical course in this case revealed that the tumor grew rapidly over a short period, highlighting the importance of carefully measuring and monitoring the tumor size.

## Supplementary Material

Suppl 1 No uterine leiomyosarcoma infiltration was observed in the ileum.

Suppl 2 Invasion of uterine leiomyosarcoma into the veins was observed within the tumor tissue of uterine leiomyosarcoma.

Suppl 3 Metastatic foci of uterine leiomyosarcoma were found in the lung tissue. Metastatic foci of uterine leiomyosarcoma are indicated by yellow dotted circles.

## Data Availability

Data are publicly available on various websites. The details are provided in the first paragraph of the Results section. Information from this clinical study and the associated transparency statements in the medical journal articles are available online. (https://kyoto.hosp.go.jp/html/guide/medicalinfo/clinicalresearch/expand/gan.html; accessed on 15 March 2025).
