# Supplementary figures and images for: Ruptured Uterine Leiomyosarcoma With Heterologous Components Including Osteosarcoma and Chondrosarcoma
Source: J Med Cases. 2026 Mar 4;17(4):157–62. doi: 10.14740/jmc5266 (PMC12978401; doi:10.14740/jmc5266)

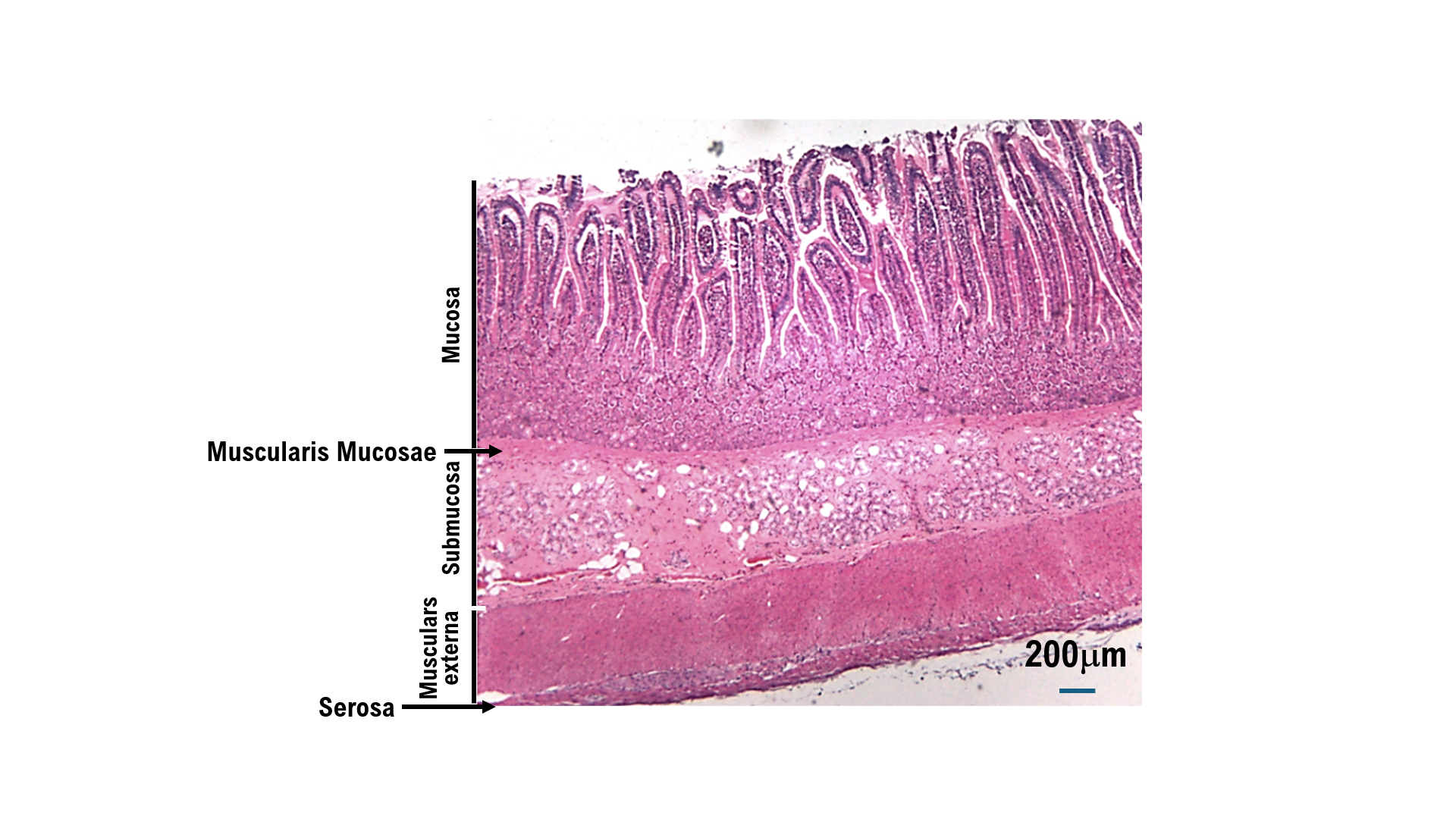


**Suppl 1.** No uterine leiomyosarcoma infiltration was observed in the ileum.

Supplement: Suppl 1 — No uterine leiomyosarcoma infiltration was observed in the ileum. [file jmc-17-04-157-s001.docx]
