# Supplementary material for: Ruptured Uterine Leiomyosarcoma With Heterologous Components Including Osteosarcoma and Chondrosarcoma
Source: J Med Cases. 2026 Mar 4;17(4):157–62. doi: 10.14740/jmc5266 (PMC12978401; doi:10.14740/jmc5266)
Supplement: Suppl 2 — Invasion of uterine leiomyosarcoma into the veins was observed within the tumor tissue of uterine leiomyosarcoma. [file jmc-17-04-157-s002.docx]

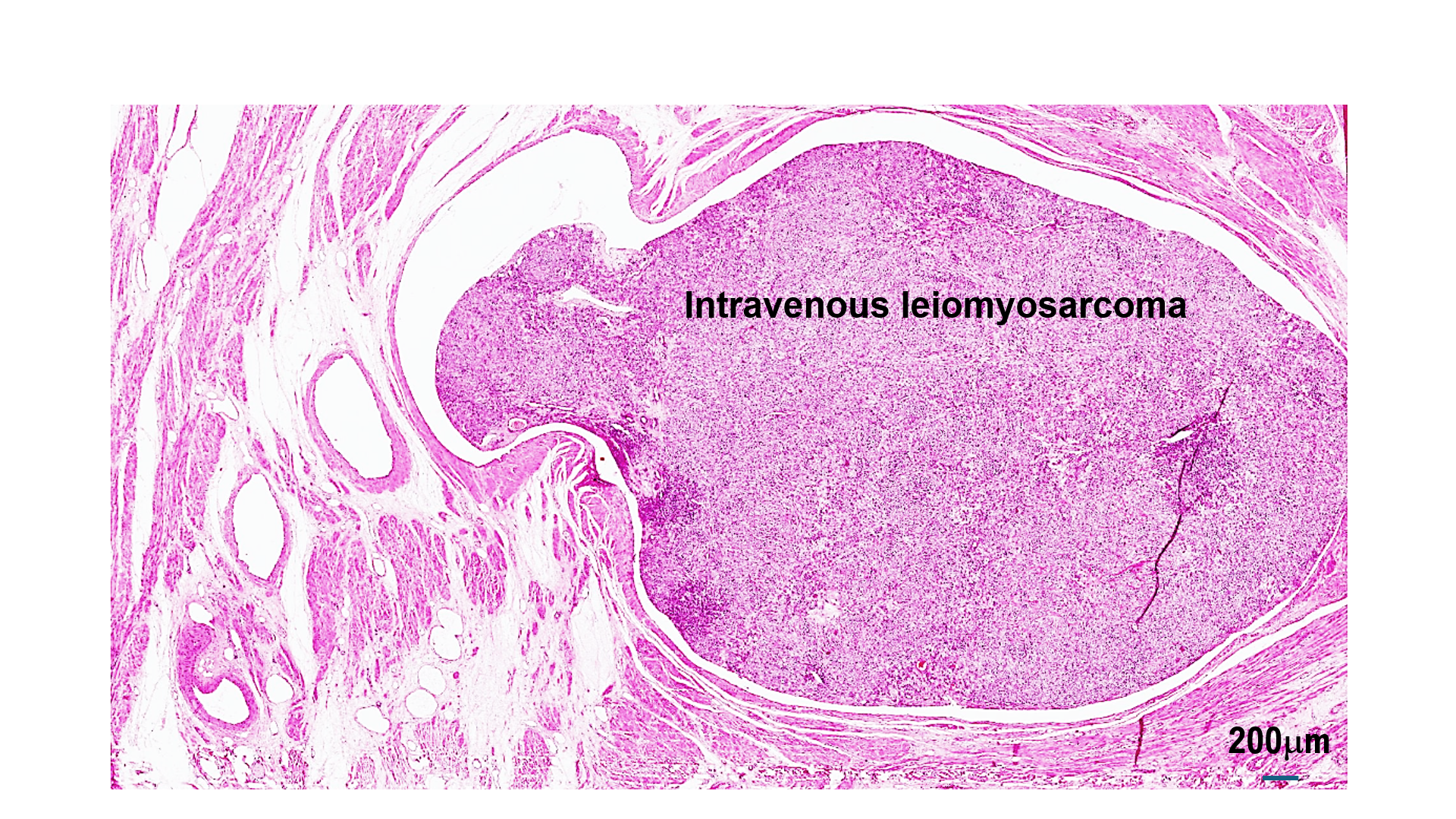


**Suppl 2.** Invasion of uterine leiomyosarcoma into the veins was observed within the tumor tissue of uterine leiomyosarcoma.
