# Supplementary material for: Ruptured Uterine Leiomyosarcoma With Heterologous Components Including Osteosarcoma and Chondrosarcoma
Source: J Med Cases. 2026 Mar 4;17(4):157–62. doi: 10.14740/jmc5266 (PMC12978401; doi:10.14740/jmc5266)
Supplement: Suppl 3 — Metastatic foci of uterine leiomyosarcoma were found in the lung tissue. Metastatic foci of uterine leiomyosarcoma are indicated by yellow dotted circles. [file jmc-17-04-157-s003.docx]

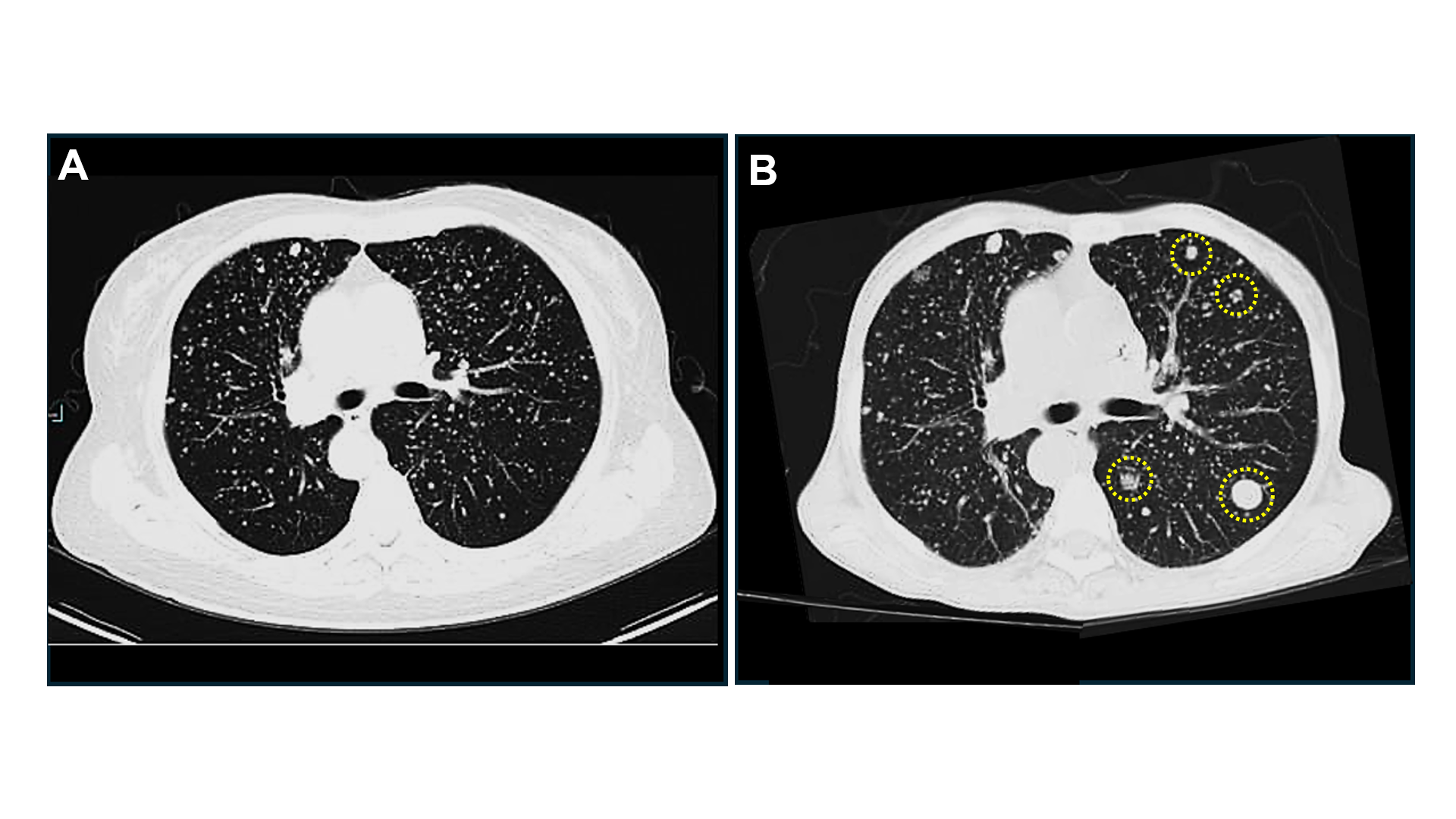


**Suppl 3.** Metastatic foci of uterine leiomyosarcoma were found in the lung tissue. Metastatic foci of uterine leiomyosarcoma are indicated by yellow dotted circles.
